# Supplementary material for: Cytotoxic Isopentenyl Phloroglucinol Compounds from Garcinia xanthochymus Using LC-MS-Based Metabolomics
Source: Metabolites. 2023 Feb 10;13(2):258. doi: 10.3390/metabo13020258 (PMC9964469; doi:10.3390/metabo13020258)
Supplement: Supplementary file 1 [file metabolites-13-00258-s001.zip › metabolites-2102681-supplementary.pdf]

Supplementary Material

Cytotoxic Isopentenyl Phloroglucinol Compounds from *Garcinia xanthochymus* Using LC-MS-Based Metabolomics

Fan Quan, Xinbo Luan, Jian Zhang, Wenjie Gao, Jian Yan * and Ping Li *

Key Laboratory of Agro-Environment in the Tropics, Ministry of Agriculture and Rural Affairs, Guang-dong Provincial Key Laboratory of Eco-Circular Agriculture, Guangdong Engineering Research Centre for Modern Eco-Agriculture, College of Natural Resources and Environment, South China Agricultural University, Guangzhou 510642, China

Table S1. Metabolic compositions were putatively identified by UPLC-QTOF-MS and MSE analysis in *G. xanthochymus.*

Peak NO.	Rt. (min)	MS molecular ions [M + H]+/ [M-H]-			MSE fragment ions			Identification [Ref.]	Class	Plant Parts
		m/z	M.F.	ppm	*m/z*	M.F.	ppm			
1	0.656	-	-	-	-	-	-	eucryphin [1]	flavonoid	leaf
		339.0726	$C_{15}H_{15}O_9$	2.9	-	-	-			
2	2.137	-	-	-	-	-	-	unknown		leaf
		549.2546	$C_{25}H_{41}O_{13}$	-0.2	371.2073	$C_{19}H_{31}O_7$	0.8			
3	2.200	-	-	-	-	-	-	xanthochymusside [2]	biflavonoid	leaf
		719.1609	$C_{36}H_{31}O_{16}$	-0.4	593.1293	$C_{30}H_{25}O_{13}$	-0.3			
					431.0757	$C_{24}H_{15}O_8$	-2.3			
					295.0246	$C_{16}H_7O_6$	1.0			
4	2.269	719.1571	$C_{36}H_{31}O_{16}$	-5.7	557.1080	$C_{30}H_{21}O_{11}$	-0.7	fukugiside [3]	biflavonoid	fruit, leaf, branch, stem bark
					431.0710	$C_{24}H_{15}O_8$	0.9			
					403.0826	$C_{23}H_{15}O_7$	2.0			
		717.1461	$C_{36}H_{29}O_{16}$	0.7	565.1332	$C_{29}H_{25}O_{12}$	-2.5			
					429.0600	$C_{24}H_{13}O_8$	-2.3			
5	2.435	721.1726	$C_{36}H_{33}O_{16}$	-6	-	-	-	xanthochymusside [2]	biflavonoid	leaf
		719.1605	$C_{36}H_{31}O_{16}$	-1	593.1277	$C_{30}H_{25}O_{13}$	-3			
					431.0753	$C_{24}H_{15}O_8$	-3.2			
					295.0243	$C_{16}H_7O_6$	0.0			
6	2.469	575.1177	$C_{30}H_{23}O_{12}$	-2.3	431.0762	$C_{24}H_{15}O_8$	-1.2	buchananiflavanone [4]	biflavonoid	leaf
					403.0818	$C_{23}H_{15}O_7$	0.0			
					297.0412	$C_{16}H_9O_6$	4.4			
		573.1037	$C_{30}H_{21}O_{12}$	0.7	447.0706	$C_{24}H_{15}O_9$	0.2			
					429.0603	$C_{24}H_{13}O_8$	-2.3			
					295.0240	$C_{16}H_7O_6$	-1.0			
7	2.653	559.1230	$C_{30}H_{23}O_{11}$	-1.8	-	-	-	GB-2a or its isomers	biflavonoid	leaf

Peak	Rt.	MS molecular ions [M + H]+/ [M-H]-			MSE fragment ions			Identification	Class	Plant Parts
NO.	(min)	*m/z*	M.F.	ppm	*m/z*	M.F.	ppm	[Ref.]		
		557.1085	$C_{30}H_{21}O_{11}$	0.2	447.0728	$C_{24}H_{15}O_{9}$	2.7	[2]		
					429.0606	$C_{24}H_{13}O_{8}$	-0.9			
8	2.734	557.1075	$C_{30}H_{21}O_{11}$	-1.6	539.0980	$C_{30}H_{19}O_{10}$	0.4	fukugetin [5] or	biflavonoid	fruit, leaf, branch,
					431.0771	$C_{24}H_{15}O_{8}$	0.9	morelloflavone [6]		stem bark, seed
					403.0822	$C_{23}H_{15}O_{7}$	1.0			
		555.0930	$C_{30}H_{19}O_{11}$	0.5	429.0601	$C_{24}H_{13}O_{8}$	-2.1			
					401.0679	$C_{23}H_{13}O_{7}$	4.5			
					295.0238	$C_{16}H_{7}O_{6}$	-1.7			
9	2.751	559.1226	$C_{30}H_{23}O_{11}$	-2.5	-	-	-	GB-1 or its isomers [7]	biflavonoid	leaf
		557.1077	$C_{30}H_{21}O_{11}$	-1.3	429.0597	$C_{24}H_{13}O_{8}$	-2.1			
					401.0652	$C_{23}H_{13}O_{7}$	-2.2			
					295.0240	$C_{16}H_{7}O_{6}$	-0.3			
10	2.917	543.1270	$C_{30}H_{23}O_{10}$	-3.9	415.0827	$C_{24}H_{15}O_{7}$	2.2	GB 1a [3]	biflavonoid	leaf, branch
					387.0878	$C_{23}H_{15}O_{6}$	2.3			
					297.0409	$C_{16}H_{9}O_{6}$	3.4			
		541.1129	$C_{30}H_{21}O_{10}$	-1.1	413.0654	$C_{24}H_{13}O_{7}$	-4.4			
					385.0700	$C_{23}H_{13}O_{6}$	-3.6			
					295.0241	$C_{16}H_{7}O_{6}$	-1.9			
11	2.917	541.1126	$C_{30}H_{21}O_{10}$	-1.7	415.0827	$C_{24}H_{15}O_{7}$	3.9	volkensiflavone [8]	biflavonoid	stem bark, seed
					387.0882	$C_{23}H_{15}O_{6}$	1.3			
		539.0979	$C_{30}H_{19}O_{10}$	0.2	429.0598	$C_{24}H_{13}O_{8}$	-2.8			
					413.0652	$C_{24}H_{13}O_{7}$	-2.2			
					385.0706	$C_{23}H_{13}O_{6}$	-1.6			
					295.0241	$C_{16}H_{7}O_{6}$	-0.7			
					267.0829	$C_{15}H_{7}O_{5}$	-1.5			

Peaks NO.	Rt. (min)	MS molecular ions [M + H]+/ [M-H]-			MSE fragment ions			Identification [Ref.]	Class	Plant Parts
		m/z	M.F.	ppm	*m/z*	M.F.	ppm			
12	2.969	539.0970	$C_{30}H_{19}O_{10}$	-1.5	415.0821	$C_{24}H_{15}O_7$	0.7	amentoflavone [9]	biflavonoid	leaf
					387.0879	$C_{23}H_{15}O_6$	2.6			
		537.0814	$C_{30}H_{17}O_{10}$	-1.5	443.0392	$C_{24}H_{11}O_9$	-2.5			
13	3.382	553.1123	$C_{31}H_{21}O_{10}$	-2.2	431.0752	$C_{24}H_{15}O_8$	-3.5	sotetsuflavone [10]	biflavonoid	leaf, branch
					403.0445	$C_{22}H_{11}O_8$	-2.2			
		551.0977	$C_{31}H_{19}O_{10}$	-0.2	429.0602	$C_{24}H_{13}O_8$	-1.9			
					331.0621	$C_{20}H_{11}O_5$	4.5			
14	3.450	-	-	-	-	-	-	-	xanthone	stem bark
		669.1609	$C_{36}H_{29}O_{13}$	0.1	409.1281	$C_{23}H_{21}O_7$	-1.5			
					391.1173	$C_{23}H_{19}O_6$	-2.3			
					259.0242	$C_{13}H_7O_6$	-0.4			
15	3.548	595.1428	$C_{30}H_{27}O_{13}$	-4	-	-	-	tiliroside [11]	flavonoid	leaf
		593.1274	$C_{30}H_{25}O_{13}$	-3.5	-	-	-			
16	3.668	311.1869	$C_{17}H_{27}O_5$	3.5	-	-	-	unknown		fruit, branch, stem bark
		309.1717	$C_{17}H_{25}O_5$	4.9	-	-	-			
17	3.864	295.1918	$C_{17}H_{27}O_4$	3	-	-	-	tanacetol A [12]	benzophenone	fruit, leaf, stem bark
		293.1763	$C_{17}H_{25}O_4$	3.4	-	-	-			
18	3.864	339.1250	$C_{20}H_{19}O_5$	5.3	283.0621	$C_{16}H_{11}O_5$	5.3	6-prenylapigenin [13]	flavonoid	stem bark
		337.1085	$C_{20}H_{17}O_5$	2.7	293.0445	$C_{17}H_9O_5$	-1.7			
					281.0456	$C_{16}H_9O_5$	2.1			
19	4.064	343.1182	$C_{19}H_{19}O_6$	5.8	287.0569	$C_{15}H_{11}O_6$	4.5	9H-xanthen-9-one, 1,4, 6-trihydroxy-5-methoxy -7-(3-me thyl-2-buten- 1-yl)-[14]	xanthone	stem bark
					215.0346	$C_{12}H_7O_4$	0.9			
		341.1033	$C_{19}H_{17}O_6$	2.3	285.0399	$C_{15}H_9O_6$	0			
20	4.099	653.1633	$C_{36}H_{29}O_{12}$	-3.2	597.1015	$C_{32}H_{21}O_{12}$	-3	garmoxanthone	xanthone	stem bark

Peaks NO.	Rt. (min)	MS molecular ions [M + H]+/ [M-H]-			MSE fragment ions			Identification [Ref.]	Class	Plant Parts .
		m/z	M.F.	ppm	*m/z*	M.F.	ppm			
		651.1494	$C_{36}H_{27}O_{12}$	-1.4	391.1173	$C_{23}H_{19}O_{6}$	-2.3	[15]		
					259.0240	$C_{13}H_{7}O_{6}$	-1.2			
21	5.028	-	-	-	-	-	-	unknown		fruit, leaf, branch, stem bark
		311.1691	$C_{13}H_{27}O_{8}$	-4.8	-	-	-			
22	5.080	301.1430	$C_{18}H_{21}O_{4}$	-3.3	-	-	-	bractebiphenyl B [16]	biphenyl	fruit, leaf, branch, stem bark
		-	-	-	-	-	-			
23	5.212	535.3640	$C_{31}H_{51}O_{7}$	0.9	557.3443	$C_{31}H_{50}O_{7}Na$	-2.0	unknown		seed
					359.2241	$C_{22}H_{31}O_{4}$	5.3			
					301.1427	$C_{18}H_{21}O_{4}$	-4.3			
					227.2026	$C_{14}H_{27}O_{2}$	6.6			
		533.3484	$C_{31}H_{49}O_{7}$	1.1	501.3204	$C_{30}H_{45}O_{6}$	-2.4			
					421.2219	$C_{23}H_{33}O_{7}$	-1.7			
24	5.332	417.2656	$C_{25}H_{37}O_{5}$	3.6	439.2458	$C_{25}H_{36}O_{5}Na$	-0.2	garcinielliptone HC or garcinielliptone HD [17]	benzofurandione	seed
					329.1763	$C_{20}H_{25}O_{4}$	3.0			
					301.1424	$C_{18}H_{21}O_{4}$	-5.3			
					275.1300	$C_{16}H_{19}O_{4}$	6.2			
					257.1180	$C_{16}H_{17}O_{3}$	0.8			
		415.2493	$C_{25}H_{35}O_{5}$	2.2	345.1709	$C_{20}H_{25}O_{5}$	2			
					303.1236	$C_{17}H_{19}O_{5}$	1.3			
					287.1289	$C_{17}H_{19}O_{4}$	2.1			
					219.0660	$C_{12}H_{11}O_{4}$	1.4			
25	5.613	517.3531	$C_{31}H_{49}O_{6}$	0.4	539.3344	$C_{31}H_{48}O_{6}Na$	-0.9	Garcihombronane L [18]	friedolanostane	seed
					359.2239	$C_{22}H_{31}O_{4}$	4.7			
					291.1612	$C_{17}H_{23}O_{4}$	5.5			

Peaks NO.	Rt. (min)	MS molecular ions [M + H]+/ [M-H]-			MSE fragment ions			Identification [Ref.]	Class	Plant Parts
		m/z	M.F.	ppm	*m/z*	M.F.	ppm			
		515.3381	$C_{31}H_{47}O_6$	1.6	403.2114	$C_{23}H_{31}O_6$	-1.7			
					289.1443	$C_{17}H_{21}O_4$	1.0			
					221.0819	$C_{12}H_{13}O_4$	2.3			
26	5.746	431.2810	$C_{26}H_{39}O_5$	3	453.2633	$C_{26}H_{38}O_5Na$	3.5	unknown		seed
		429.2654	$C_{26}H_{37}O_5$	3	317.1391	$C_{18}H_{21}O_5$	0.6			
					301.1445	$C_{18}H_{21}O_4$	1.7			
					233.0817	$C_{13}H_{13}O_4$	1.3			
27	5.963	465.2289	$C_{28}H_{33}O_6$	2.6	409.1667	$C_{24}H_{25}O_6$	3.9	garciniaxanthone E [19]	xanthone	stem bark
					353.1041	$C_{20}H_{17}O_6$	4.5			
					311.0562	$C_{17}H_{11}O_6$	1.9			
		463.2126	$C_{28}H_{31}O_6$	1.1	407.1485	$C_{24}H_{23}O_6$	-2.5			
					365.1017	$C_{21}H_{17}O_6$	-2.2			
28	6.027	523.2562	$C_{27}H_{39}O_{10}$	3.6	479.2293	$C_{25}H_{35}O_9$	2.5	unknown		fruit, branch, stem bark
					435.2028	$C_{23}H_{31}O_8$	2.1			
					301.2417	$C_{18}H_{21}O_4$	-4.3			
		-	-	-	-	-	-			
29	6.095	485.2914	$C_{29}H_{41}O_6$	2.3	507.2731	$C_{29}H_{40}O_6Na$	1.6	9H-Xanthen-9-one, 1,3-dihydroxy-7-methoxy-2,8-bis(3-methylbutyl)-6-(pentyloxy)-	xanthone	fruit, branch, stem bark
		-	-	-	-	-	-			
30	6.526	517.3532	$C_{31}H_{49}O_6$	0.6	539.3342	$C_{31}H_{48}O_6Na$	-1.3	garcihombronane L [18]	friedolanostane	seed
					375.2182	$C_{22}H_{31}O_5$	2.9			
					291.1610	$C_{17}H_{23}O_4$	4.8			
					219.1032	$C_{13}H_{15}O_3$	5.0			

Peaks NO.	Rt. (min)	MS molecular ions [M + H]+/ [M-H]-			MSE fragment ions			Identification [Ref.]	Class	Plant Parts
		m/z	M.F.	ppm	*m/z*	M.F.	ppm			
		515.3384	$C_{31}H_{47}O_6$	2.1	403.2116	$C_{23}H_{31}O_6$	-1.2			
					289.1446	$C_{17}H_{21}O_4$	2.1			
					247.0977	$C_{14}H_{15}O_4$	2.8			
31	6.595	487.3430	$C_{30}H_{47}O_5$	1.4	509.3241	$C_{30}H_{46}O_5Na$	-0.4	garciosaterpene E or its		seed
					363.2181	$C_{21}H_{31}O_5$	2.8	isomers [20]		
					277.1455	$C_{17}H_{23}O_3$	2.9			
					219.1034	$C_{13}H_{15}O_3$	5.9			
		485.3279	$C_{30}H_{45}O_5$	2.5	373.2012	$C_{22}H_{29}O_5$	-0.8			
					275.1290	$C_{16}H_{19}O_4$	2.5			
					233.0820	$C_{13}H_{13}O_4$	2.6			
32	6.628	503.3373	$C_{30}H_{47}O_6$	0	-	-	-	garcinielliptone E [21]	triterpenoid	leaf, branch, seed
		501.3220	$C_{30}H_{45}O_6$	0.8	389.1955	$C_{22}H_{29}O_6$	-2.3			
					275.1284	$C_{16}H_{19}O_4$	0.4			
					233.0810	$C_{13}H_{13}O_4$	-1.7			
33	6.646	503.3378	$C_{30}H_{47}O_6$	1	525.3163	$C_{30}H_{46}O_6Na$	-5.5	garcinielliptone C [22]	phloroglucinol	seed
		501.3222	$C_{30}H_{45}O_6$	1.2	347.1860	$C_{20}H_{27}O_5$	0.6			
					289.1444	$C_{17}H_{21}O_4$	1.4			
34	6.692	515.3376	$C_{31}H_{47}O_6$	0.6	537.3181	$C_{31}H_{46}O_6NaC$	-2	cholesta-8(14),24-dien-	phloroglucinol	seed
					343.1928	$_{21}H_{27}O_4$	5.5	26-oic acid, 9,23-		
					325.1813	$C_{21}H_{25}O_3$	2.8	dihydroxy-4,4,17-		
					271.1345	$C_{17}H_{19}O_3$	4.1	trimethyl-3,15-dioxo-,		
		513.3221	$C_{31}H_{45}O_6$	1	485.3255	$C_{30}H_{45}O_5$	-2.5	methyl ester, (5α,13α,		
					359.1856	$C_{21}H_{27}O_5$	-0.6	17α,23R,24E)-(9CI)		
					289.1443	$C_{17}H_{21}O_4$	1.0	[23]		
35	6.744	465.2286	$C_{28}H_{33}O_6$	1.9	-	-	-	isogarciniaxanthone E	xanthone	stem bark

Peaks NO.	Rt. (min)	MS molecular ions [M + H]+/ [M-H]-			MSE fragment ions			Identification [Ref.]	Class	Plant Parts
		m/z	M.F.	ppm	*m/z*	M.F.	ppm			
		463.2128	$C_{28}H_{31}O_6$	1.5	351.0858	$C_{20}H_{15}O_6$	-3.1	[24]		
					323.0550	$C_{18}H_{11}O_6$	-1.9			
36	6.944	485.3291	$C_{30}H_{45}O_5$	4.9	507.3085	$C_{30}H_{44}O_5Na$	-0.2	garcinielliptone L [25]	phloroglucinol	seed
		483.3117	$C_{30}H_{43}O_5$	1.4	415.2477	$C_{25}H_{35}O_5$	-1.7	or garsubelline A [26]		
					355.1906	$C_{22}H_{27}O_4$	-0.8			
					287.1285	$C_{17}H_{19}O_4$	0.7			
					259.0974	$C_{15}H_{15}O_4$	1.5			
37	6.996	485.3223	$C_{30}H_{45}O_5$	-9.1	-	-	-	garcinielliptone F [27]	phloroglucinol	fruit, leaf, branch,
		483.3112	$C_{30}H_{43}O_5$	1.2	329.1752	$C_{20}H_{25}O_4$	-0.3			stem bark, seed
					287.1291	$C_{17}H_{19}O_4$	2.8			
					219.0658	$C_{12}H_{11}O_4$	0.5			
38	7.191	501.3572	$C_{31}H_{49}O_5$	-1.6	-	-	-	garcinielliptone D [25]	phloroglucinol	fruit, seed
		499.3426	$C_{31}H_{47}O_5$	0.6	361.2006	$C_{21}H_{29}O_5$	-2.5			
					289.1443	$C_{17}H_{21}O_4$	1.0			
39	7.294	517.3530	$C_{31}H_{49}O_6$	0.2	539.3336	$C_{31}H_{48}O_6Na$	-2.4	garcihombronane L	friedolanostane	leaf, branch, stem bark
					375.2172	$C_{22}H_{31}O_5$	0.3	[18]		
					291.1611	$C_{17}H_{23}O_4$	5.2			
		515.3376	$C_{31}H_{47}O_6$	0.6	403.2110	$C_{23}H_{31}O_6$	-2.7			
					289.1440	$C_{17}H_{21}O_4$	0.0			
					247.0969	$C_{14}H_{15}O_4$	-0.4			
40	7.478	499.3422	$C_{31}H_{47}O_5$	-0.2	-	-	-	garcinielliptone T [18]	phloroglucinol	fruit, leaf, branch,
		497.3266	$C_{31}H_{45}O_5$	-0.2	343.1904	$C_{21}H_{27}O_4$	-1.5			stem bark, seed
					301.1440	$C_{18}H_{21}O_4$	0.0			
41	7.558	499.3433	$C_{31}H_{47}O_5$	2	521.3253	$C_{31}H_{46}O_5Na$	1.9	garsubellin B [28]	phloroglucinol	stem bark, seed
					359.2243	$C_{22}H_{31}O_4$	5.8			

Peaks NO.	Rt. (min)	MS molecular ions [M + H]+/ [M-H]-			MSE fragment ions			Identification [Ref.]	Class	Plant Parts
		m/z	M.F.	ppm	*m/z*	M.F.	ppm			
		497.3269	$C_{31}H_{45}O_5$	0.4	429.2637	$C_{26}H_{37}O_5$	-0.9			
					369.2065	$C_{23}H_{29}O_4$	-0.3			
					301.1442	$C_{18}H_{21}O_4$	0.7			
					273.1132	$C_{16}H_{17}O_4$	1.8			
42	7.925	499.3423	$C_{31}H_{47}O_5$	0	521.3232	$C_{31}H_{46}O_5Na$	-2.1	garsubellin B [28]	phloroglucinol	stem bark
		497.3275	$C_{31}H_{45}O_5$	1.6	369.2065	$C_{23}H_{29}O_4$	-0.3			
					301.1446	$C_{18}H_{21}O_4$	2.0			
					233.0818	$C_{13}H_{13}O_4$	1.7			
43	8.023	483.3117	$C_{30}H_{43}O_5$	1.4	505.2931	$C_{30}H_{42}O_5Na$	0.2	unknown		seed
					329.1767	$C_{20}H_{25}O_4$	4.3			
		481.2955	$C_{30}H_{41}O_5$	0.2	-	-	-			
44	8.310	485.3267	$C_{30}H_{45}O_5$	0	-	-	-	unknown		seed
		483.3115	$C_{30}H_{43}O_5$	1	345.1703	$C_{20}H_{25}O_5$	0.3			
					303.1237	$C_{17}H_{19}O_5$	1.6			
45	8.660	497.3270	$C_{31}H_{45}O_5$	0.6	519.3076	$C_{31}H_{44}O_5Na$	-1.9	unknown		seed
					343.1917	$C_{21}H_{27}O_4$	2.3			
					325.1812	$C_{21}H_{25}O_3$	2.5			
		495.3113	$C_{31}H_{43}O_5$	0.6	-	-	-			
46	8.907	499.3428	$C_{31}H_{47}O_5$	1	521.3243	$C_{31}H_{46}O_5Na$	0	garsubellin E [28]	phloroglucinol	seed
		497.3276	$C_{31}H_{45}O_5$	1.8	359.1858	$C_{21}H_{27}O_5$	0			
					317.1793	$C_{18}H_{21}O_5$	1.3			
					275.1285	$C_{16}H_{19}O_4$	0.7			
47	9.039	411.2916	$C_{27}H_{39}O_3$	4.1				garcinal [29]	tocotrienol	seed
		-	-	-	-	-	-			

References of Table S1

1. Yoshimura, M.; Ninomiya, K.; Tagashira, Y.; Maejima, K.; Yoshida, T.; Amakura, Y. Polyphenolic Constituents of the Pericarp of Mangosteen (*Garcinia Mangostana* L.). *J. Agric. Food. Chem.* **2015,** 65, 7670-7674.
2. Li, Y.; Chen, Y.; Xiao, C.; Chen, D.; Xiao, Y.; Mei, Z. Rapid Screening and Identification of α-Amylase Inhibitors from *Garcinia Xanthochymus* Using Enzyme-Immobilized Magnetic Nanoparticles Coupled with HPLC and MS. *J. Chromatogr. B.* **2014,** 960, 166-173.
3. Pandey, R.; Chandra, P.; Kumar, B. G. Y. C.; Srivastva, M.; Anu Aravind, A. P.; Shameer, P. S.; Rameshkumar, K. B. Simultaneous Determination of Multi-Class Bioactive Constituents for Quality Assessment of *Garcinia* Species Using UHPLC-QQQLIT-MS/MS. *Ind. Crop. Prod.* **2015,** 77, 861-872.
4. Aemail, T. D. S. C.; Ranner, J.; Stiglbauer, B.; Weiss, P.; Stark, S.; Balemba, O. B.; Hofmann, T. Construction and Application of a Database for a Five-Dimensional Identification of Natural Compounds in Garcinia Species by Means of UPLC-ESI-TWIMS-TOF-MS: Introducing Gas Phase Polyphenol Conformer Drift Time Distribution Intensity Ratios. *J. Agric. Food. Chem.* **2019,** 67, 975-985.
5. Chen, Y.; Gan, F.; Jin, S.; Liu, H.; Wu, S. J.; Yang, W. T.; Yang, G. Z. Adamantyl Derivatives and Rearranged Benzophenones from *Garcinia Xanthochymus* Fruits. *RSC. Adv.* **2017,** 7, 17289-17296.
6. Aravind, A. P. A.; Pandey, R.; Kumar, B.; Asha, K. R. T.; Rameshkumar, K. B. Phytochemical Screening of Garcinia Travancorica by Hplc-Esi-Qtof Mass Spectrometry and Cytotoxicity Studies of the Major Biflavonoid Fukugiside. *Nat. Prod. Commun.* **2016,** 11, 1839-1842.
7. Baslas, R. K.; Kumar, P. Chemical Examination of the Fruits of *Garcinia Xanthochymus*. *Curr. Sci.* **1979,** 48, 814-815.
8. Liu, L.; Li, Y. F.; Gan, F.; Yang, G. Z.; Chen, Y. Chemical Constituents from Leaves of *Garcinia Xanthochymus*. *China J. Chin. Mater. Med.* **2016,** 41, 2098-2104.
9. Li, P.; Yue, G. G. L.; Kwok, H. F.; Long, C. L.; Lau, C. B. S.; Kennelly, E. J. Using Ultra-Performance Liquid Chromatography Quadrupole Time of Flight Mass Spectrometry-Based Chemometrics for the Identification of Anti-Angiogenic Biflavonoids from Edible Garcinia Species. *J. Agric. Food. Chem.* **2017,** 65, 8348-8355.
10. Parveen, N.; Singh, M. P.; Khan, N. U.; Logani, M. K. Flavonoidic Constituents of *Garcinia Xanthochymus* Leaves. *Fitoterapia* **1994,** 65, 89.
11. Quan, G. H.; Oh, S. R.; Kim, J. H.; Lee, H. K.; Kinghorn, A. D.; Chin, Y. W. Xanthone Constituents of the Fruits of *Garcinia Mangostana* with Anticomplement Activity. *Phytother. Res.* **2010,** 24, 1575-1577.
12. Shiekh, K. A.; Benjakul, S.; Sae-Leaw, T. Effect of Chamuang (*Garcinia Cowa* Roxb.) Leaf Extract on Inhibition of Melanosis and Quality Changes of Pacific White Shrimp During Refrigerated Storage. *Food. Chem.* **2019,** 270, 554-561.
13. Han, Q. B.; Qiao, C. F.; Song, J. Z.; Yang, N. Y.; Cao, X. W.; Peng, Y.; Yang, D. J.; Chen, S. L.; Xu, H. X. Cytotoxic Prenylated Phenolic

Compounds from the Twig Bark of *Garcinia Xanthochymus*. *Chem. Biodivers.* **2007,** 4, 940-946.
14. Song, J. L.; Gao, H.; Yuan, L.; Wang, Z. L. Study on the Chemical Constituents of *Garcinia Xanthochymus*. *J. Anhui. Agric. Sci.* **2015,** 43, 222-224.
15. Wang, W. Y.; Liao, Y. Y.; Huang, X. M.; Tang, C.; Cai, P. A Novel Xanthone Dimer Derivative with Antibacterial Activity Isolated from the Bark of *Garcinia Mangostana*. *Nat. Prod. Res.* **2018,** 32, 1769-1774.
16. Xu, X. M.; Shi, J. L.; Li, L.; Zhu, D. L.; Yu, Z. H.; Yu, W. L.; Zhou, M.; Hu, Q. F.; Guo, Y. D.; Lou, J. Biphenyls from the Twigs of *Garcinia Multiflora* and Their Antitobacco Mosaic Virus Activities. *Rec. Nat. Prod.* **2016,** 10, 566-571.
17. Lin, K. W.; Huang, A. M.; Tu, H. Y.; Weng, J. R.; Hour, T. C.; Wei, B. L.; Yang, S. C.; Wang, J. P.; Pu, Y. S.; Lin, C. N. Phloroglucinols Inhibit Chemical Mediators and Xanthine Oxidase, and Protect Cisplatin-Induced Cell Death by Reducing Reactive Oxygen Species in Normal Human Urothelial and Bladder Cancer Cells. *J. Agric. Food. Chem.* **2009,** 57, 8782-8787.
18. Klaiklay, S.; Sukpondma, Y.; Rukachaisirikul, V.; Phongpaichit, S. Friedolanostanes and Xanthones from the Twigs of *Garcinia Hombroniana*. *Phytochemistry* **2013,** 85, 161-166.
19. Zhong, F. F.; Chen, Y.; Wang, P.; Feng, H. J.; Yang, G. Z. Xanthones from the Bark of *Garcinia Xanthochymus* and Their 1,1-Diphenyl-2-Picrylhydrazyl Radical-Scavenging Activity. *Chinese. J. Chem.* **2009,** 27, 74-80.
20. Pailee, P. A.; Kruahong, T. B.; Hongthong, S. A.; Kuhakarn, C. A.; Jaipetch, T. A.; Pohmakotr, M. A.; Jariyawat, S. C.; Suksen, K. C.; Akkarawongsapat, R. D.; Limthongkul, J. D.; Panthong, A. E.; Kongsaeree, P. A.; Prabpai, S. A.; Tuchinda, P. A.; Reutrakul, V. A. Cytotoxic, Anti-Hiv–1 and Anti-Inflammatory Activities of Lanostanes from Fruits of *Garcinia Speciosa*. *Phytochem. Lett.* **2017,** 20, 111-118.
21. Grossman, R. B.; Yang, X. W. Structural Revision of Garcinielliptin Oxide and Garcinielliptone E. *J. Nat. Prod.* **2020,** 83, 2041-2044.
22. Weng, J. R.; Lin, C. N.; Tsao, L. T.; Wang, J. P. Novel and Anti-Inflammatory Constituents of *Garcinia Subelliptica*. *Chem. Eur. J.* **2003,** 9, 1958-1963.
23. Vieira, L. M. M.; Kijjoa, A.; Wilairat, R.; Nascimento, M.; Gales, L.; Damas, A. M.; Silva, A. M. S.; Mondranondra, I. O.; Herz, W. Bioactive Friedolanostanes and 11(10→8)-Abeolanostanes from the Bark of *Garcinia Speciosa*. *J. Nat. Prod.* **2004,** 3, 2043-2047.
24. Ji, F.; Li, Z. L.; Niu, S. L.; Hua, H. M. Studies on the Chemical Constituents of the Barks of *Garcinia Xanthochymus*. *Chin. J. Med. Chem.* **2012,** 22, 507-510.
25. Weng, J. R.; Tsao, L. T.; Wang, J. P.; Wu, R. R.; Lin, C. N. Anti-Inflammatory Phloroglucinols and Terpenoids from *Garcinia Subelliptica*. *J. Nat. Prod.* **2004,** 67, 1796-1799.
26. Lin, K. W.; Huang, A. M.; Yang, S. C.; Weng, J. R.; Hour, T. C.; Pu, Y. S.; Lin, C. N. Cytotoxic and Antioxidant Constituents from *Garcinia*

Subelliptica. *Food. Chem.* **2012,** 135, 851-859.

27. Yun, Y. S.; Shioura, M.; Hitotsuyanagi, Y.; Yotsumoto, S.; Inoue, H. Garcinielliptone G from *Garcinia Subelliptica* Induces Apoptosis in Acute Leukemia Cells. *Molecules.* **2021,** 26, 2422.
28. Fukuyama, Y.; Minami, H.; Kuwayama, A. Garsubellins, Polyisoprenylated Phloroglucinol Derivatives from *Garcinia Subelliptica*. *Phytochemistry* **1998,** 49, 853-857.
29. Okoko, T. In Vitro Antioxidant and Free Radical Scavenging Activities of Garcinia Kola Seeds. *Food. Chem. Toxicol.* **2009,** 47, 2620-2623.

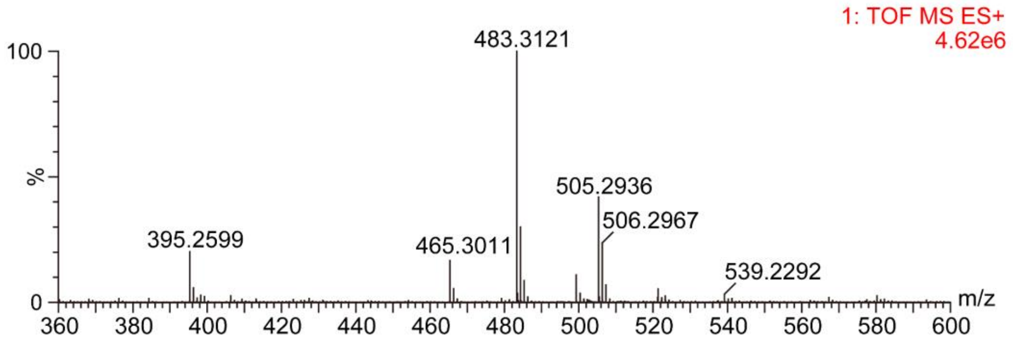

Figure S1. HR-ESI-MS spectrum for Garxanthochin A.

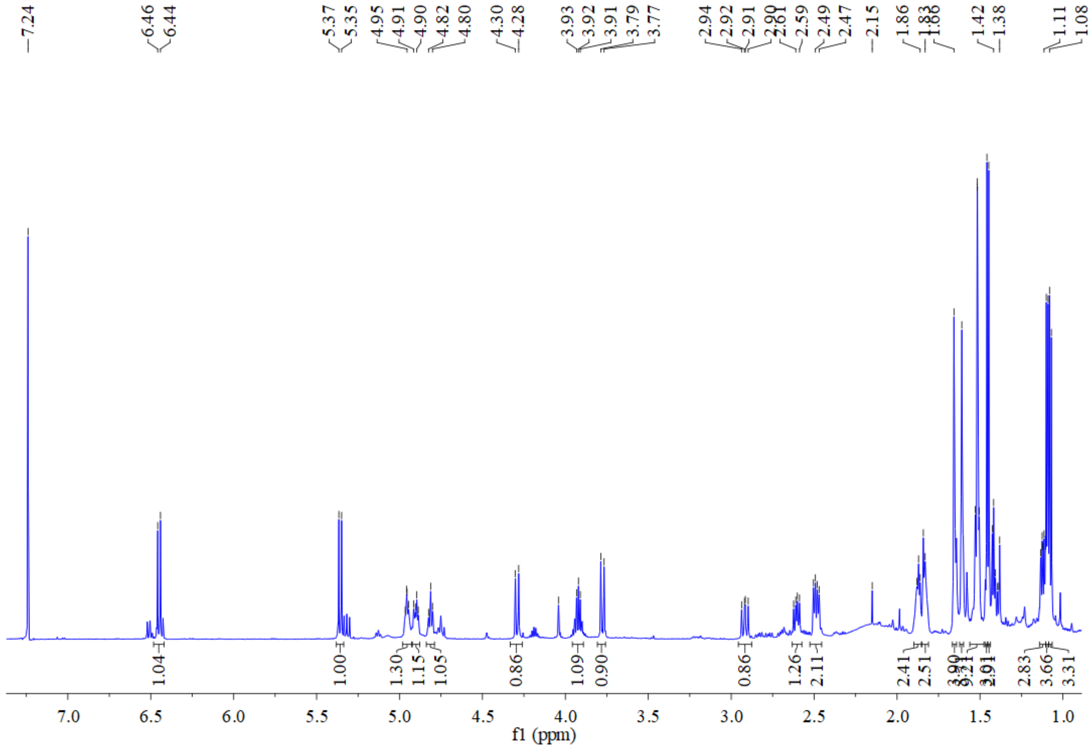

Figure S2. ^{1}H NMR spectrum for Garxanthochin A in $CDCl_3$ (600 MHz).

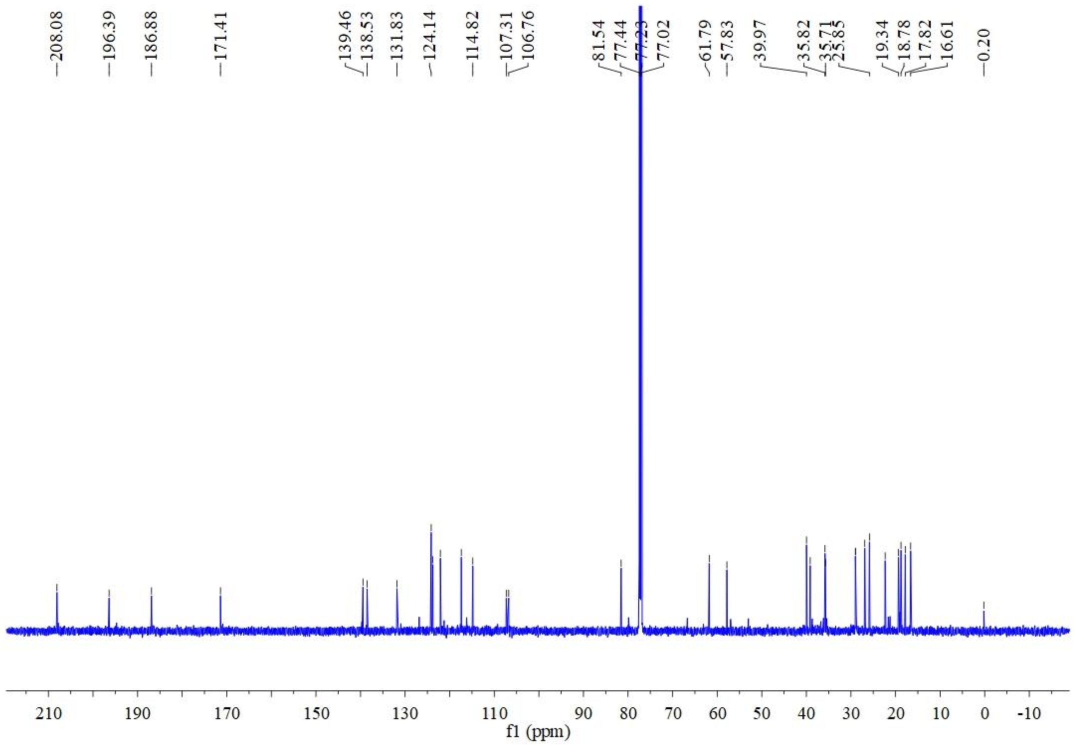

Figure S3. ^{13}C NMR spectrum for Garxanthochin A in $CDCl_3$ (150 MHz).

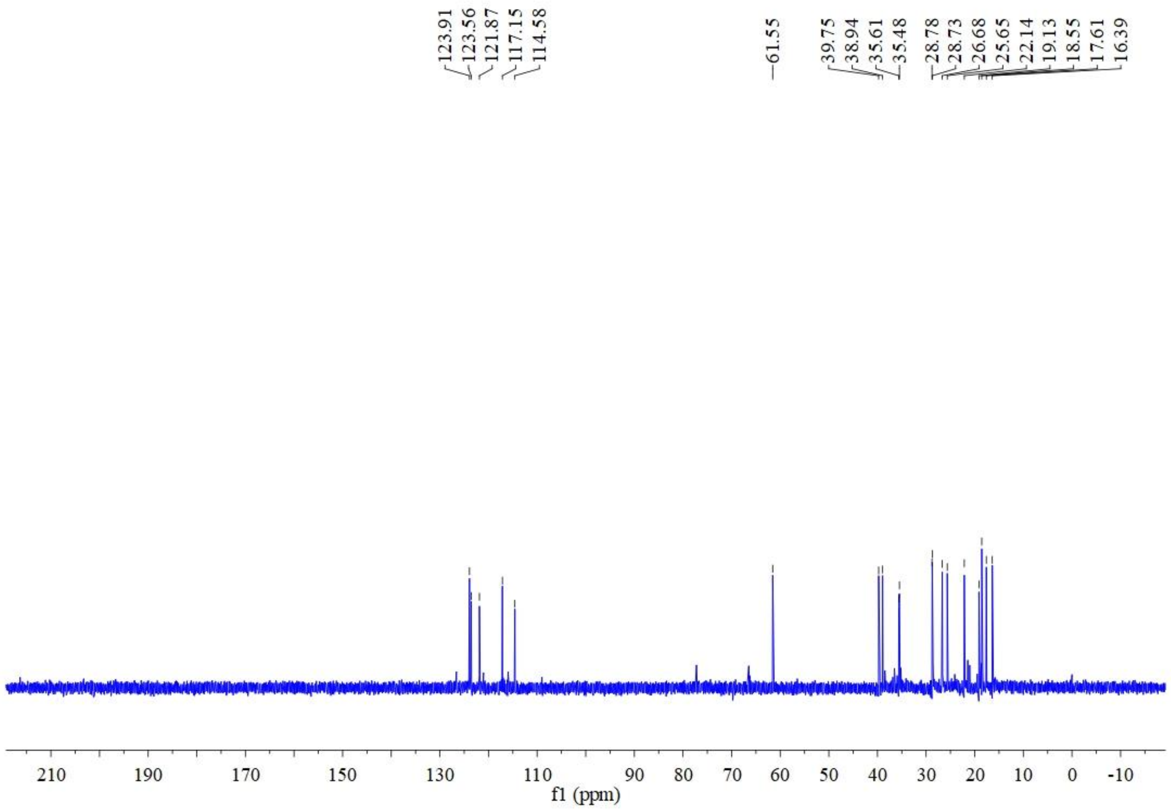

Figure S4. DEPT NMR spectrum for Garxanthochin A in $CDCl_3$ (150 MHz).

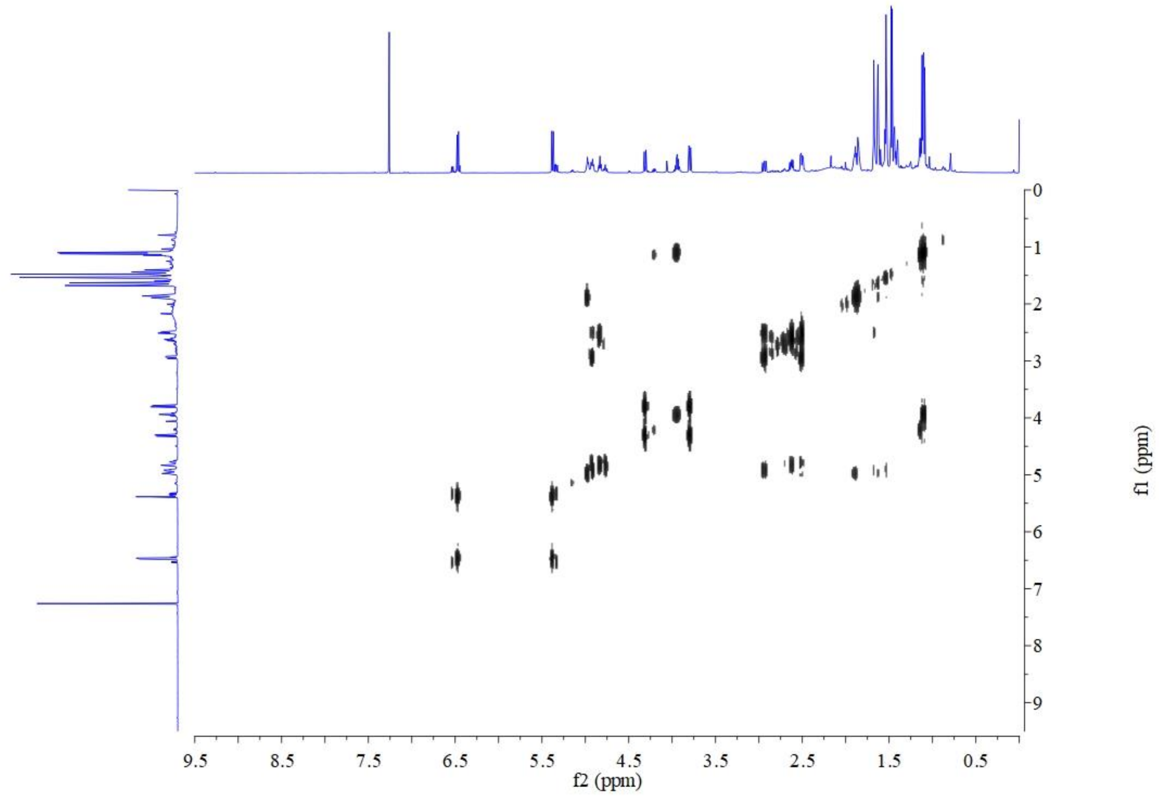

Figure S5. ^{1}H-^{1}H COSY spectrum for Garxanthochin A in $CDCl_3$.

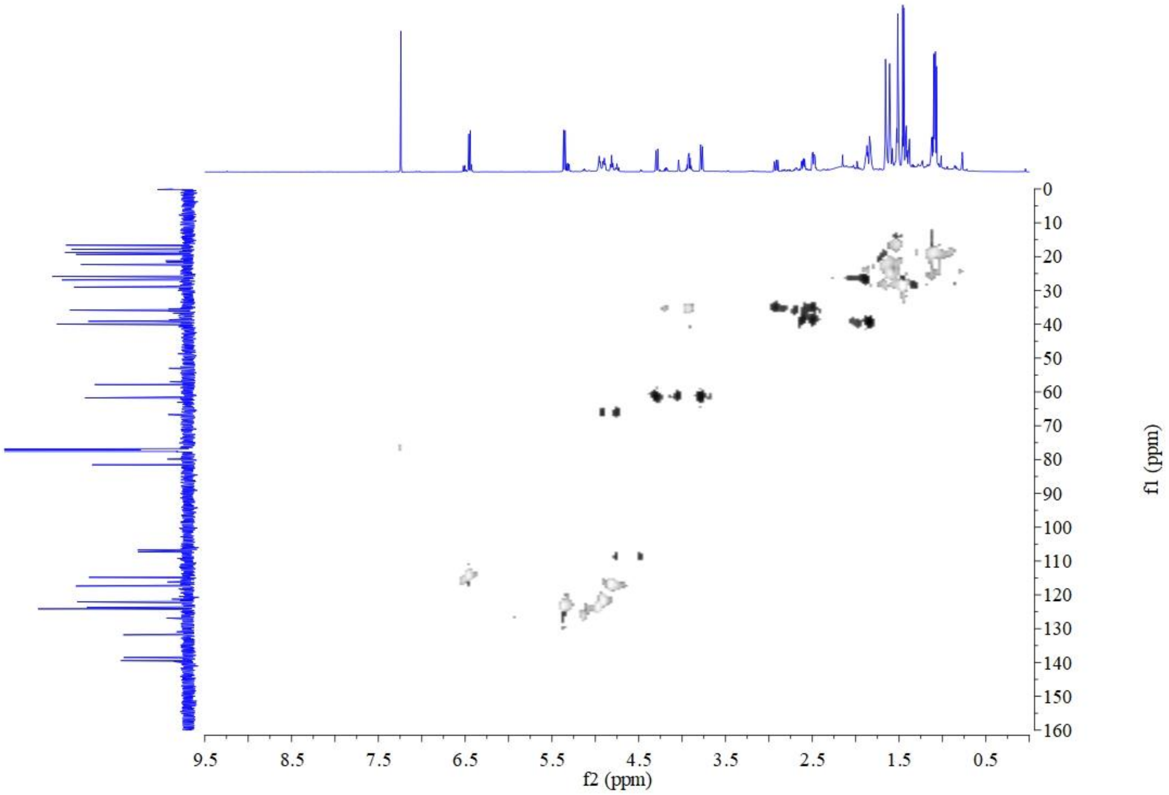

Figure S6. HMQC spectrum for Garxanthochin A in $CDCl_3$.

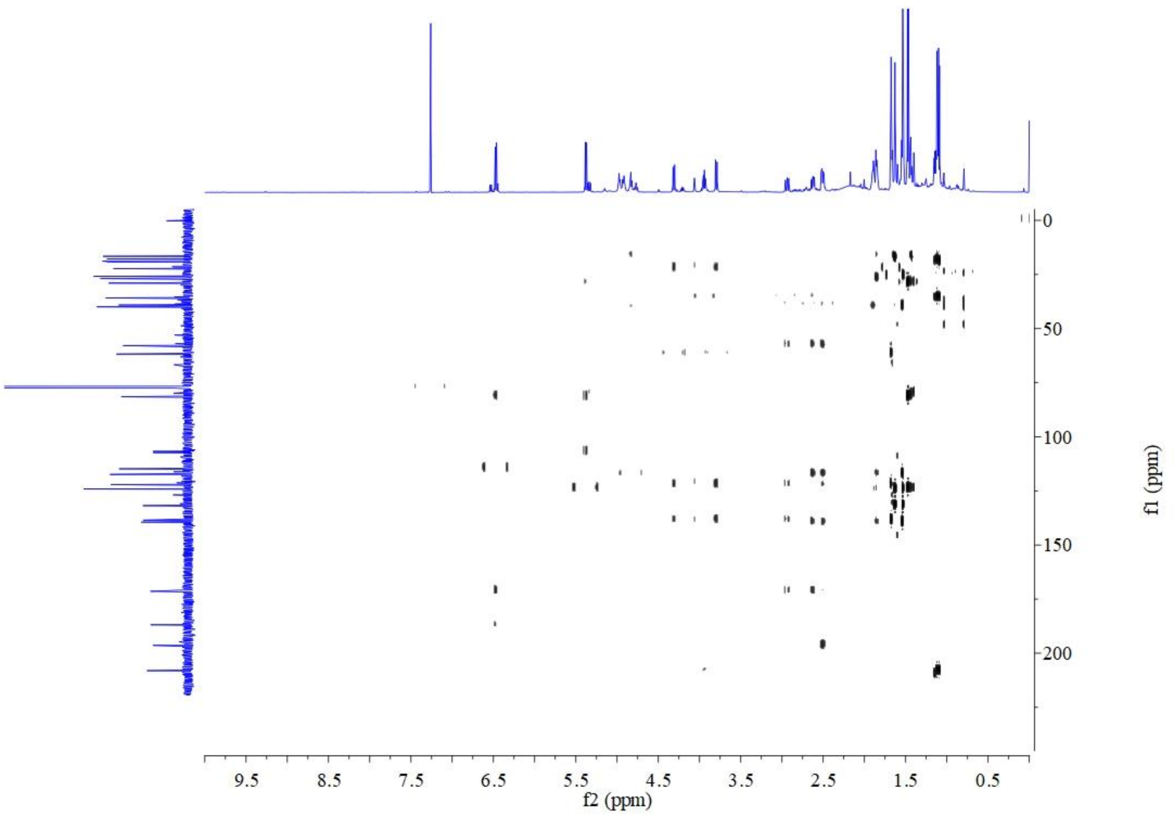

Figure S7. NOESY spectrum for Garxanthochin A in $CDCl_3$.

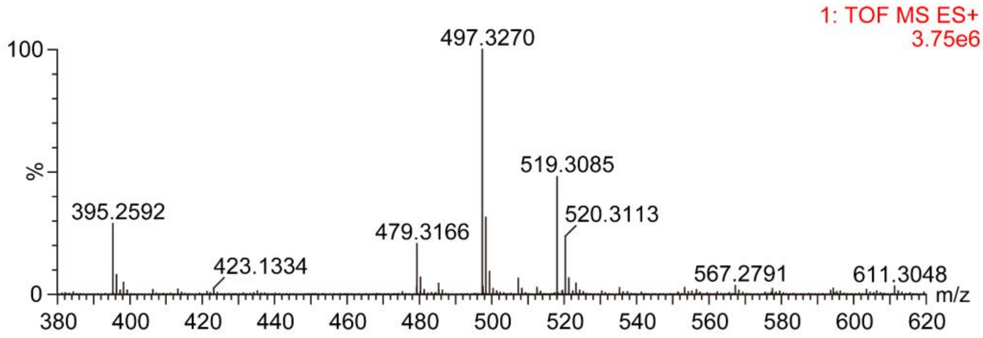

Figure S8. HR-ESI-MS spectrum for Garxanthochin B.

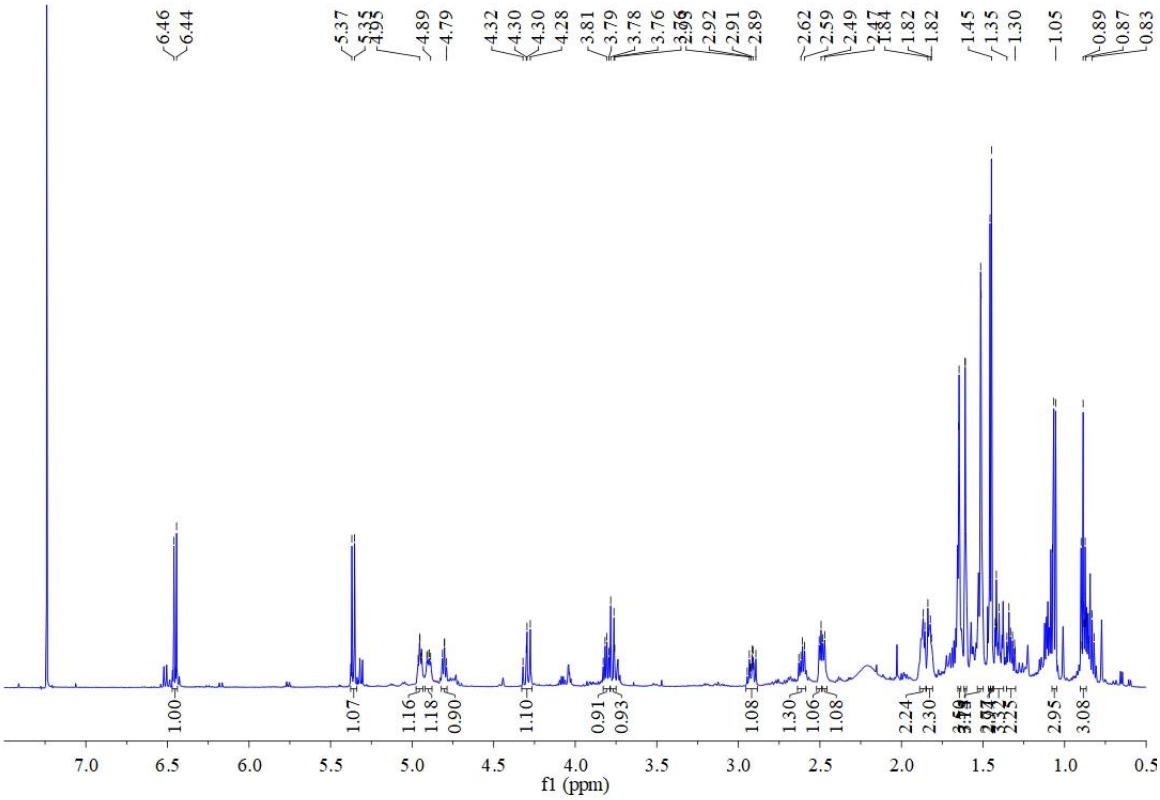

Figure S9. ^{1}H NMR spectrum for Garxanthochin B in $CDCl_3$ (600 MHz).

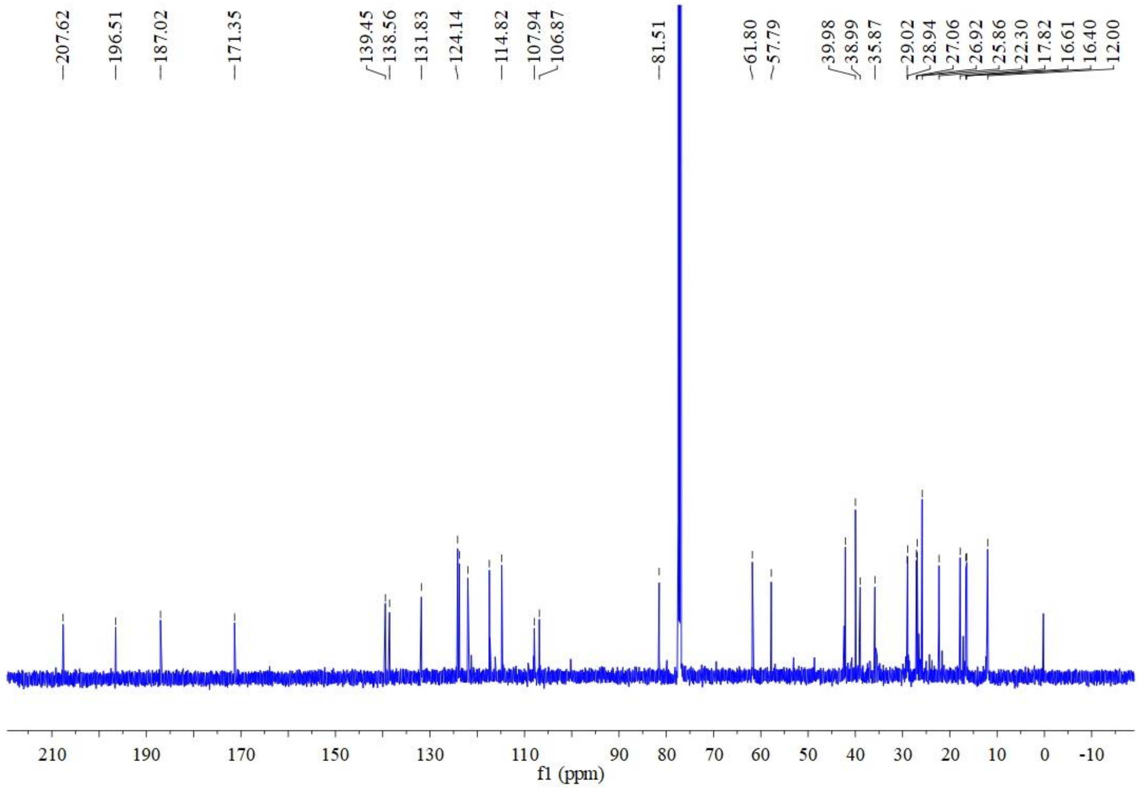

Figure S10. ^{13}C NMR spectrum for Garxanthochin B in $CDCl_3$ (150 MHz).

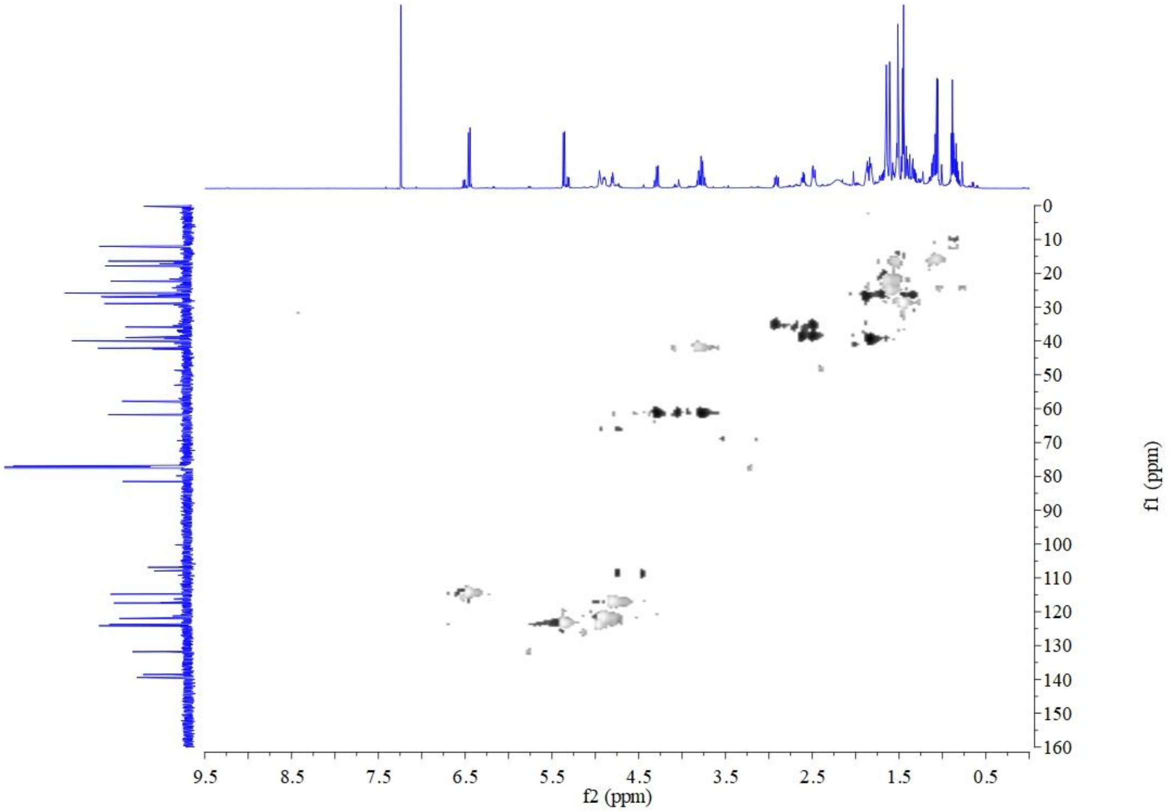

Figure S11. HMQC spectrum for Garxanthochin B in $CDCl_3$.

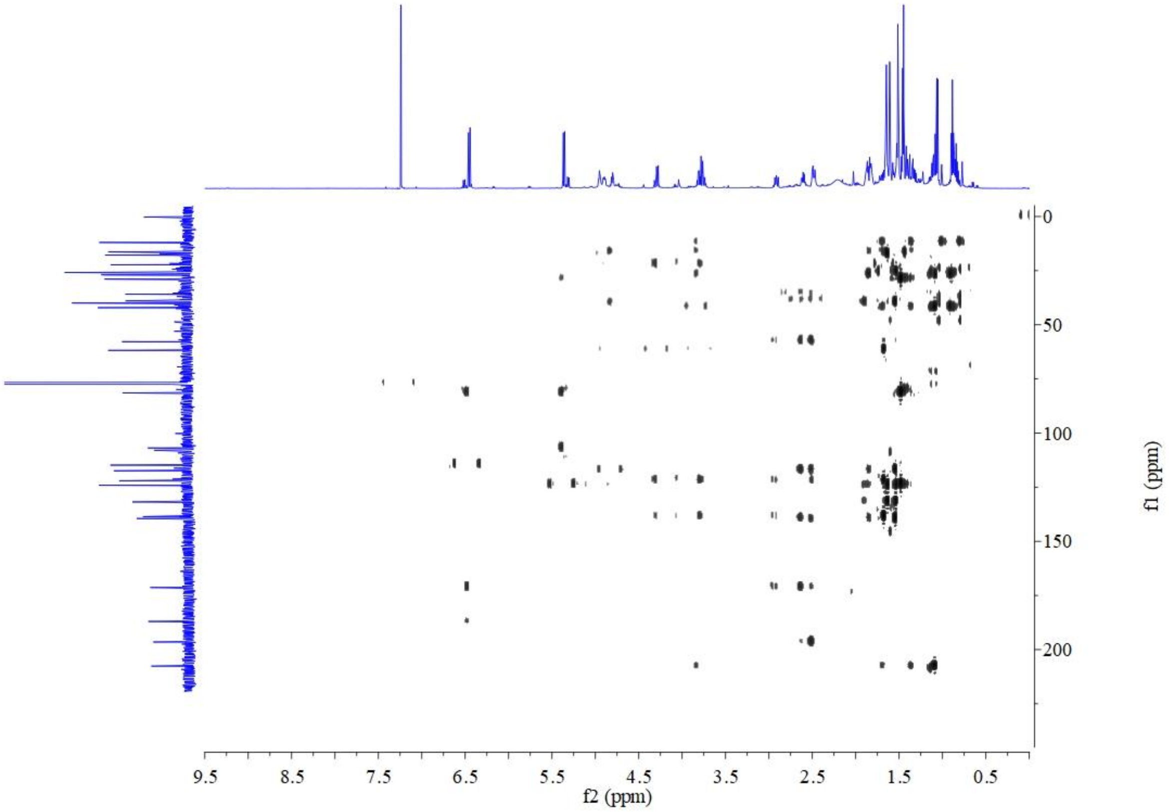

Figure S12. HMBC spectrum for Garxanthochin B in $CDCl_3$.

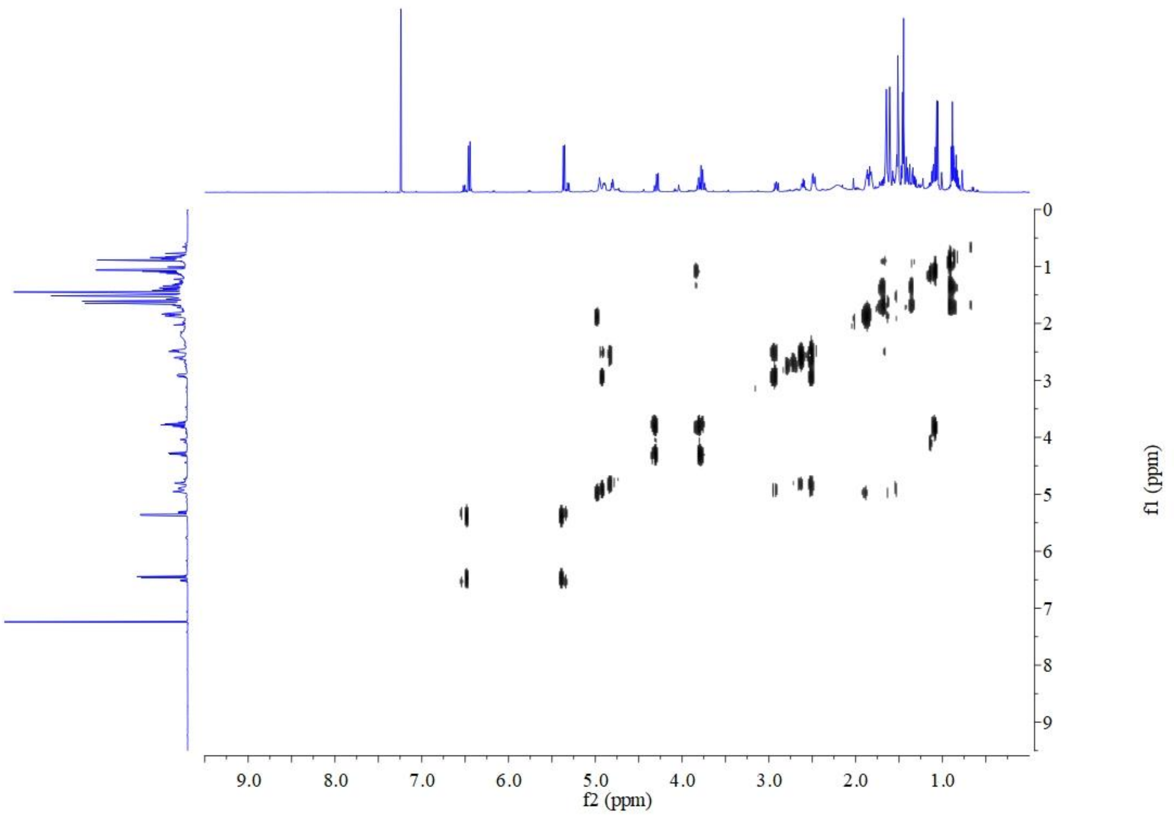

Figure S13. ¹H-¹H COSY spectrum for Garxanthochin B in $CDCl_3$.

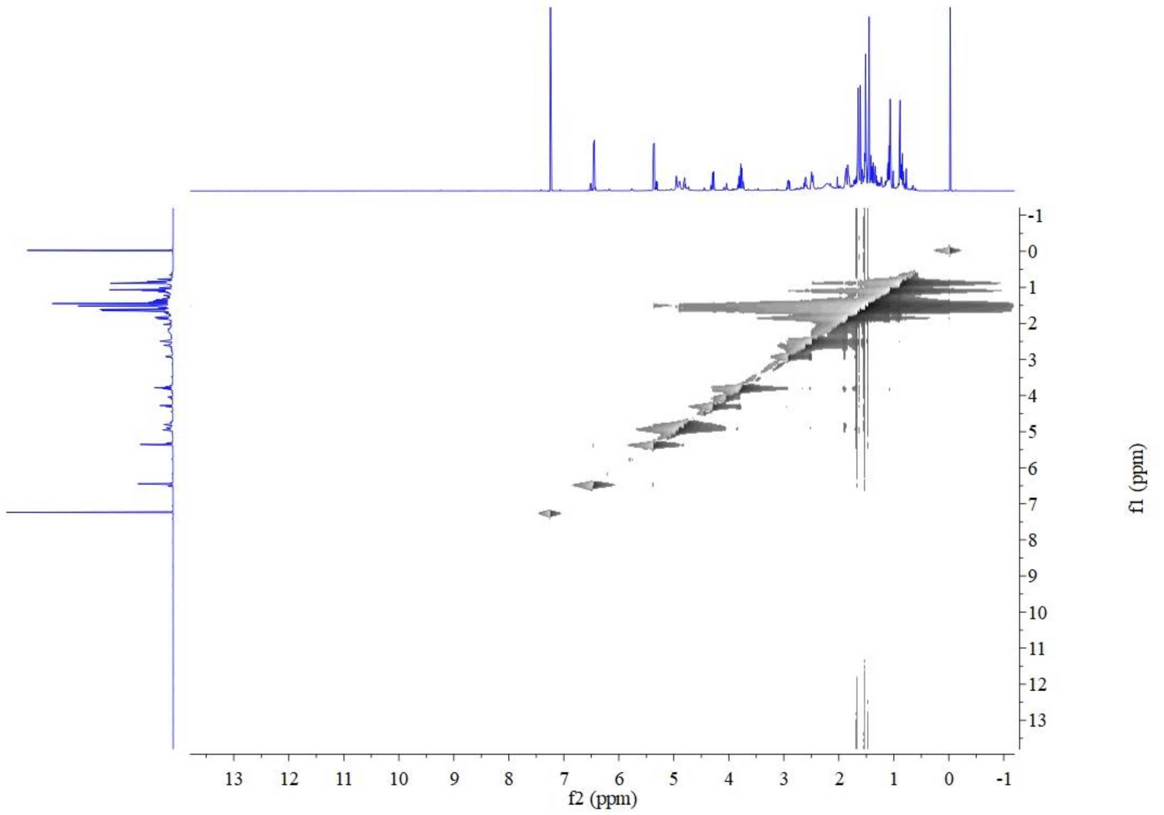

Figure S14. NOESY spectrum for Garxanthochin B in $CDCl_3$.

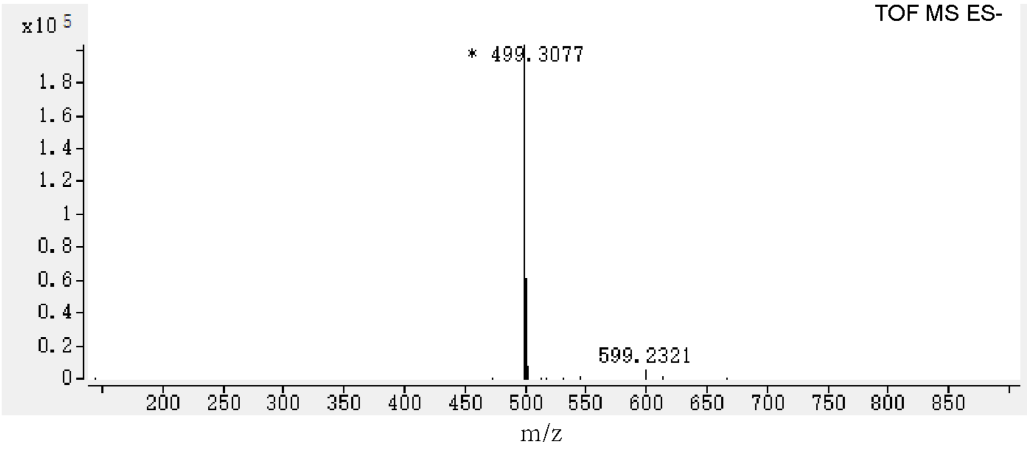

Figure S15. HR-ESI-MS spectrum for Garxanthochin C.

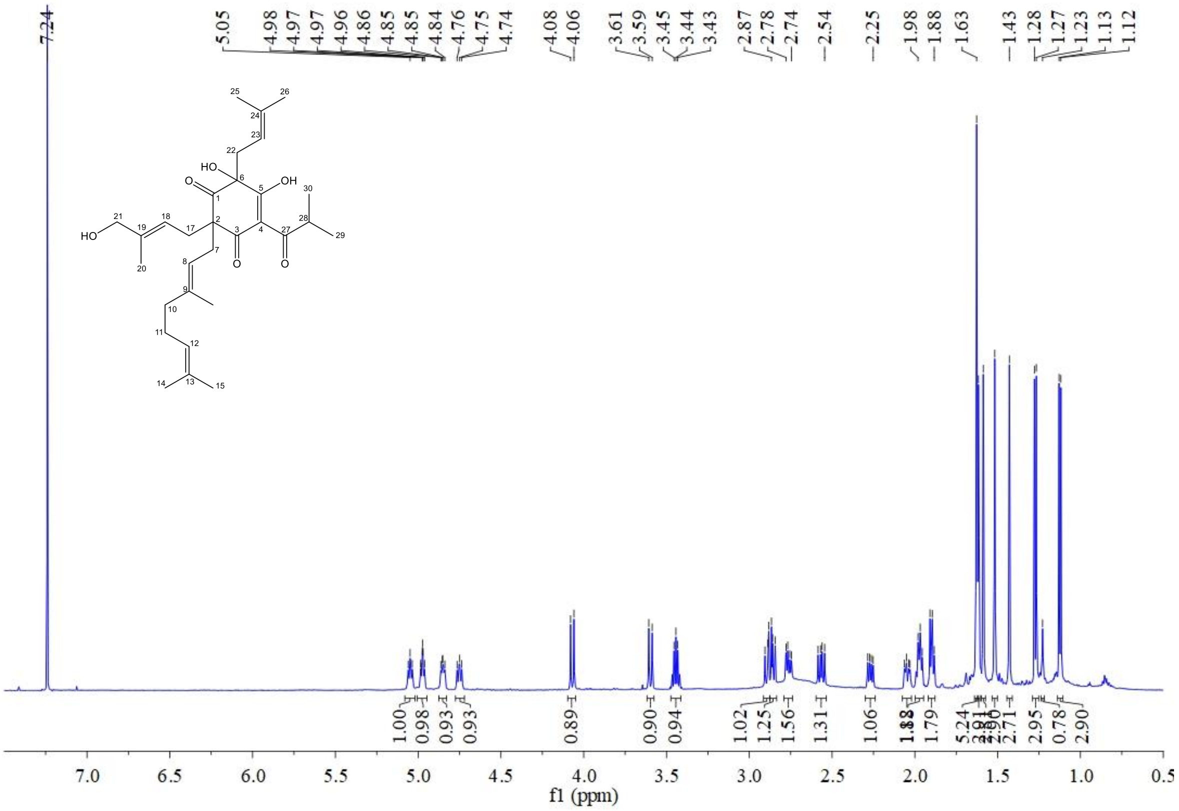

Figure S16. ^{1}H NMR spectrum for Garxanthochin C in $CDCl_3$ (600 MHz).

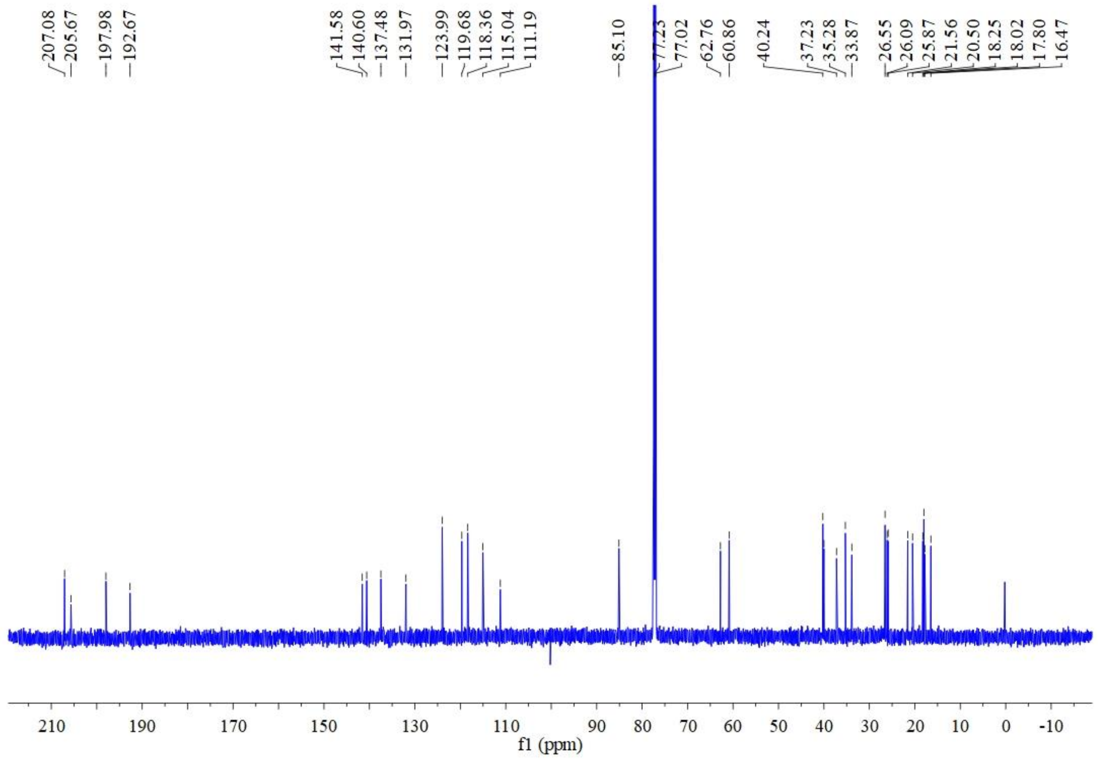

Figure S17. ^{13}C NMR spectrum for Garxanthochin C in $CDCl_3$ (150 MHz).

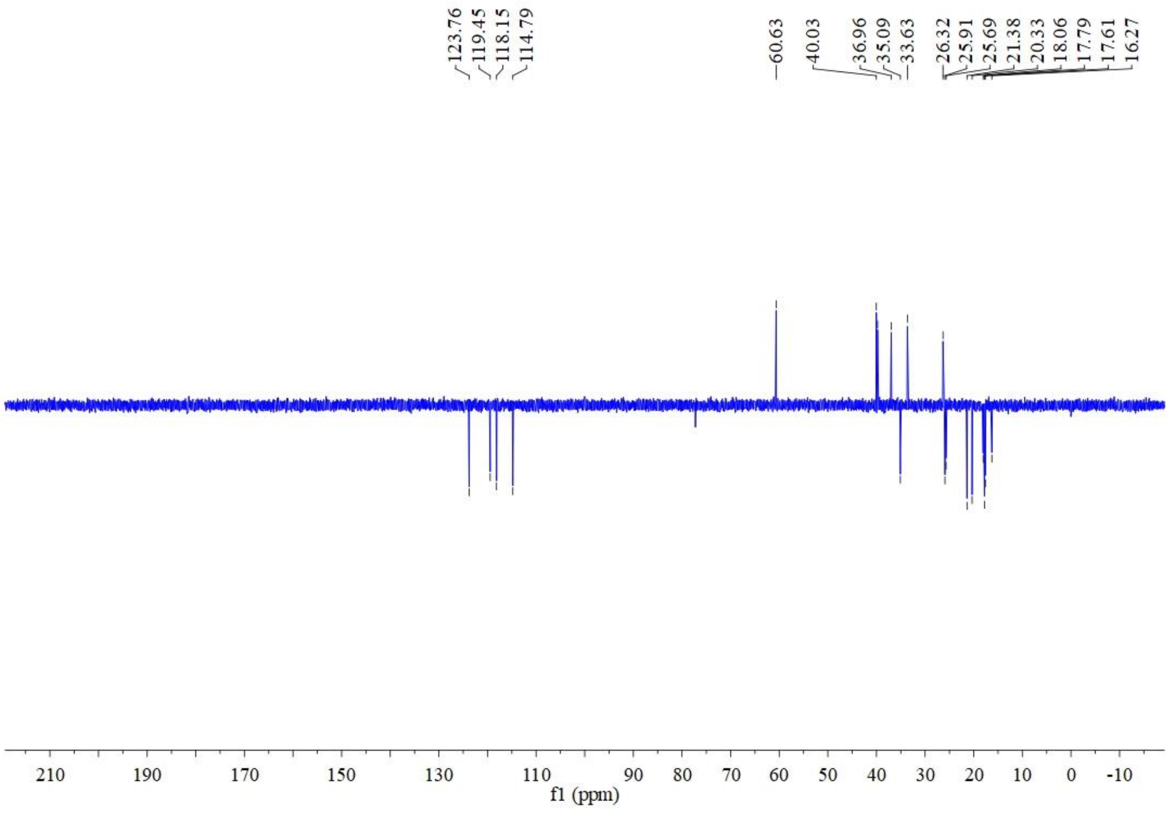

Figure S18. DEPT NMR spectrum for Garxanthochin C in $CDCl_3$ (150 MHz).

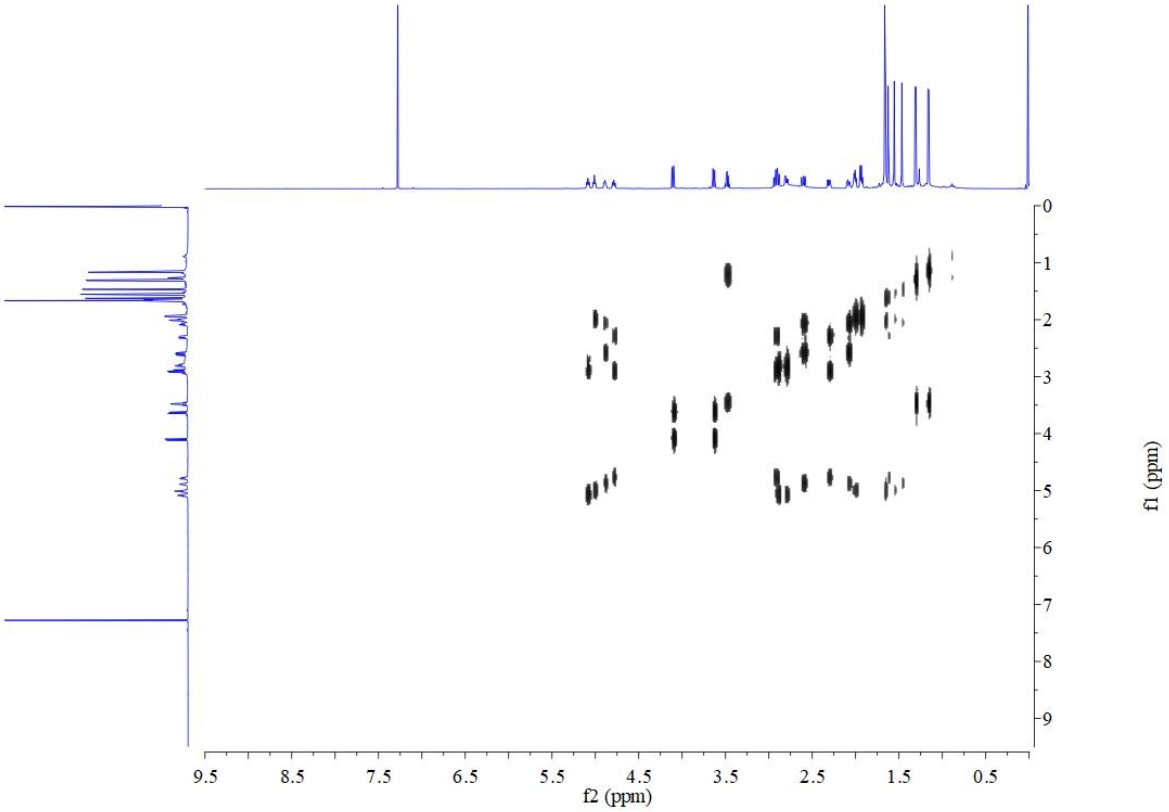

Figure S19. ^{1}H-^{1}H COSY spectrum for Garxanthochin C in $CDCl_3$.

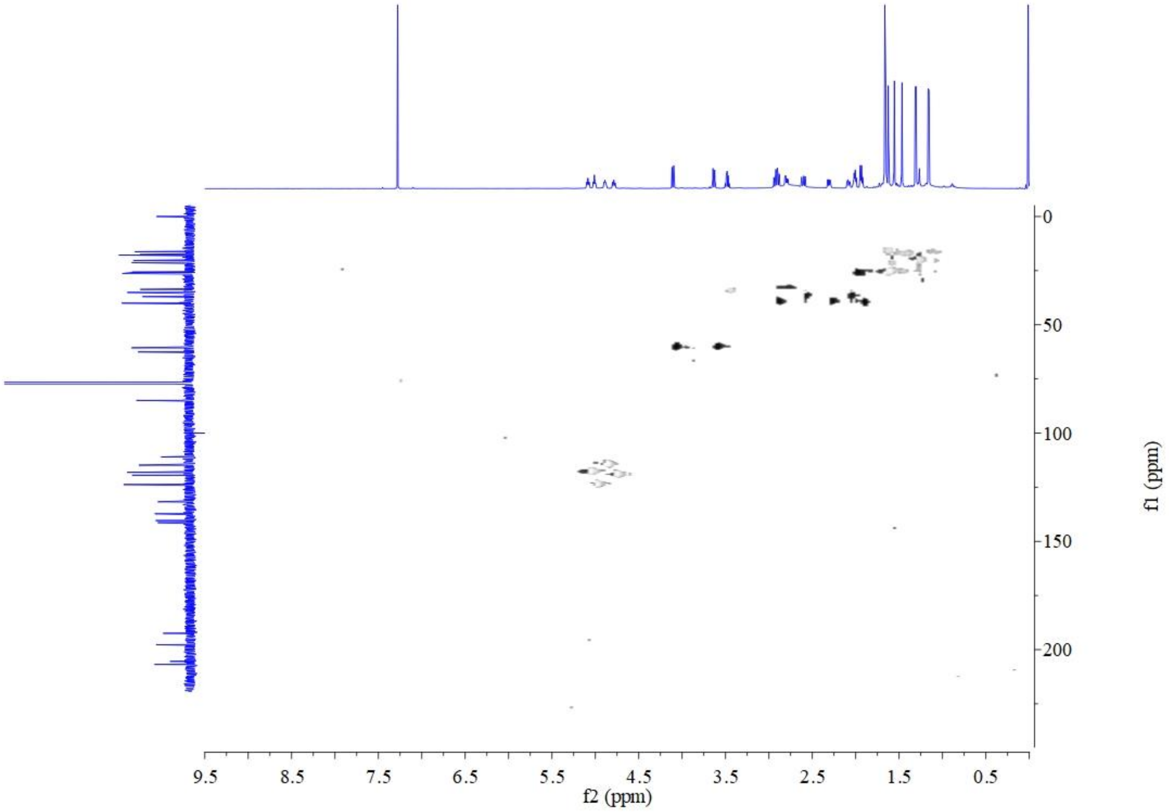

Figure S20. HMQC spectrum for Garxanthochin C in $CDCl_3$.

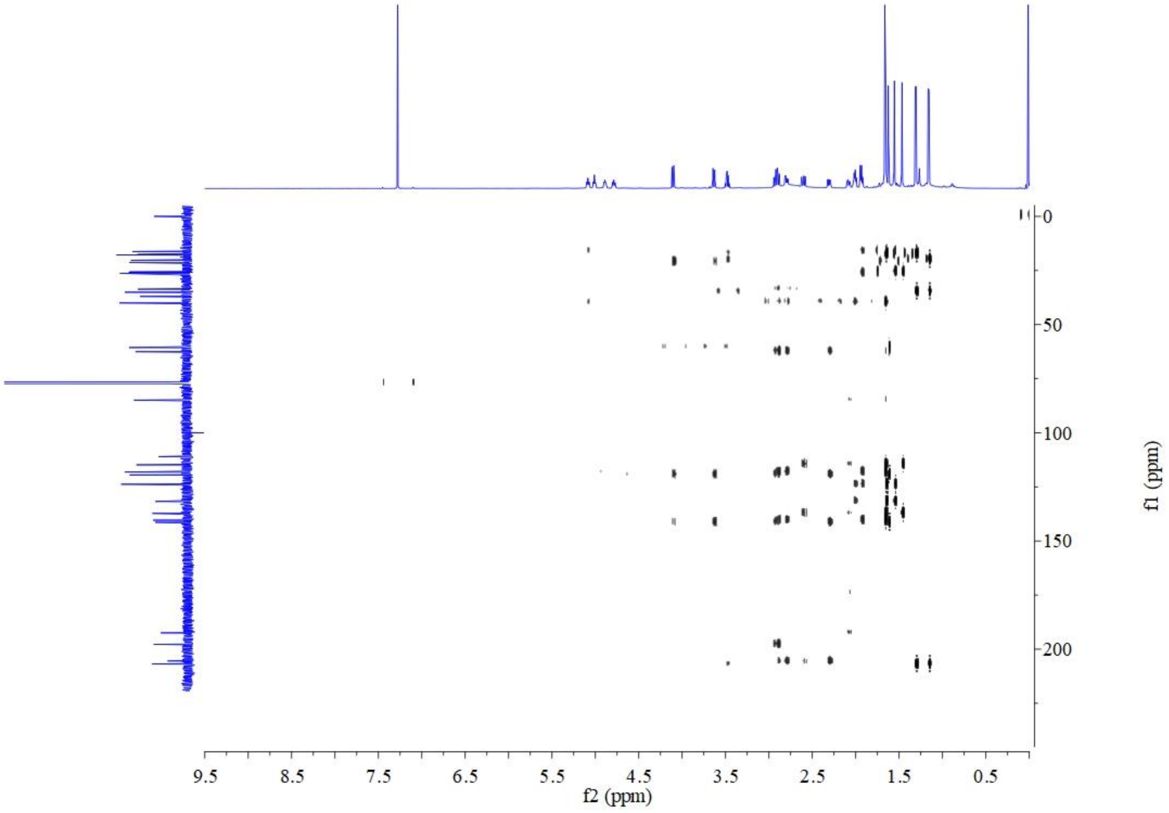

Figure S21. HMBC spectrum for Garxanthochin C in $CDCl_3$.
